# Reducing maritime accidents in ships by tackling human error: a bibliometric review and research agenda

**DOI:** 10.1186/s41072-021-00098-y

**Published:** 2021-11-24

**Authors:** Carine Dominguez-Péry, Lakshmi Narasimha Raju Vuddaraju, Isabelle Corbett-Etchevers, Rana Tassabehji

**Affiliations:** 1 Grenoble INP*, CERAG, Univ. Grenoble Alpes, 38000 Grenoble, France; 2 Visiting Fellow at the University of Bath School of Management, Bath, UK

**Keywords:** Ship accident, Human error, Socio-technical use of information technologies, Organisation, Bibliometric review

## Abstract

Over the past decade the number of maritime transportation accidents has fallen. However, as shipping vessels continue to increase in size, one single incident, such as the oil spills from ‘super’ tankers, can have catastrophic and long-term consequences for marine ecosystems, the environment and local economies. Maritime transport accidents are complex and caused by a combination of events or processes that might ultimately result in the loss of human and marine life, and irreversible ecological, environmental and economic damage. Many studies point to direct or indirect human error as a major cause of maritime accidents, which raises many unanswered questions about the best way to prevent catastrophic human error in maritime contexts. This paper takes a first step towards addressing some of these questions by improving our understanding of upstream maritime accidents from an organisation science perspective—an area of research that is currently underdeveloped. This will provide new and relevant insights by both clarifying how ships can be described in terms of organisations and by considering them in a whole ecosystem and industry. A bibliometric review of extant literature of the causes of maritime accidents related to human error was conducted, and the findings revealed three main root causes of human and organisational error, namely, human resources and management, socio-technical Information Systems and Information Technologies, and individual/cognition-related errors. As a result of the bibliometric review, this paper identifies the gaps and limitations in the literature and proposes a research agenda to enhance our current understanding of the role of human error in maritime accidents. This research agenda proposes new organisational theory perspectives—including considering ships as organisations; types of organisations (highly reliable organisations or self-organised); complex systems and socio-technical systems theories for digitalised ships; the role of power; and developing dynamic safety capabilities for learning ships. By adopting different theoretical perspectives and adapting research methods from social and human sciences, scholars can advance human error in maritime transportation, which can ultimately contribute to addressing human errors and improving maritime transport safety for the wider benefit of the environment and societies ecologies and economies.

## Introduction

The global shipping industry is responsible for transporting as much as 90% of world trade (SSR 2021). Over the past decade, improved ship design, technology, regulation and risk management systems have contributed to a 70% drop in reported shipping losses (SSR 2021). However, while the frequency of maritime accidents may be in decline, one single incident can have catastrophic and long-term consequences for marine ecosystems, the environment and local economies (Roberts et al. 2002). This is exacerbated further by the fact that maritime transportation vessels are increasing in size and the amounts of cargo on-board with them. For instance, in September 2019, Brazil’s north-eastern state of Bahia declared an emergency after an oil spill from the tanker Bouboulina contaminated kilometres of coastal beaches. In August 2020, Mauritius also declared a state of environmental emergency after the MV Wakashio ran aground at Pointe d’Esny, spilling oil into an area renowned as a sanctuary for rare wildlife. These types of accidents attract the attention of the media and heighten the concerns of people around the world, as images of the damage to marine wildlife and the environment are graphically visible.

Despite the ostensible fall in total reported losses, the number of accidents^1^, especially those related to passenger/car carrier vessels and ro-ros has increased, as has the number of reported casualties (SSR 2021). Therefore, this study's starting point was to understand further why maritime accidents with such wide-ranging consequences continue to occur.

Maritime transport accidents are complex (Guven-Koçak 2015) and caused by a combination of events or processes (Soares and Teixeira 2001) involving various actors that ultimately lead to disastrous consequences including loss of human and marine life and irreparable ecological, environmental and economic damage (Harrald et al. 1998). Apart from uncontrollable acts of God defined as ‘an extreme interruption with a natural cause (e.g. earthquake, storm, etc.)’ (Kristiansen 2005:14), the literature consistently highlights human error (HE) as one of the main contributing factors in more than 85% of cases of maritime accidents (Acejo et al. 2018; Galieriková, 2019). Furthermore, experts estimate that 30–50% of oil spills are caused directly or indirectly by HE (Michel and Fingas 2016). Despite this, there is a surprising dearth of research in the management literature investigating HE in the maritime context (Berkowitz et al. 2019). This leads us to question the role of humans in the maritime transport ecosystem and ask: ‘*What is the current state-of-the-art research regarding human error as the main cause of maritime transportation accidents? How have researchers considered and framed human error? What research agenda is recommended to integrate the “human” further to avoid human error from an organisation science perspective, including team, organisational and collaborative networks/ecosystems?*

This paper aims to address these questions by improving our understanding of maritime accidents and prevention from an organisational perspective, which is currently underdeveloped in organisation science. In order to achieve these objectives, a bibliometric review is conducted. The bibliometric review (BR) is a quantitative approach that uses co-citation analysis to visualise the literature in the field (van Oorschot et al. 2018). This reduces the reviewers’ subjectivity and bias and will generate a more systematic and encompassing picture of HE research in the field of maritime transportation.

The paper is organised as follows. The first part lays out the general context of the maritime transportation industry, the main causes of vessel accidents and the role of HE in maritime accidents. Then the five-step bibliometric review method adopted for this study is described. The findings are collated, analysed and discussed to provide a deeper understanding of what currently constitutes HE. Finally, a research agenda to investigate maritime accidents and HE from a socio-organisational perspective to prevent future accidents is proposed.

## Accidents in maritime shipping

The maritime transportation industry’s distinct maritime culture is characterised by its global nature, working conditions, autonomy and complexity (Güven-Koçak 2015). The global nature of the shipping industry means worldwide competition is driving ship-owners to seek ever-increasing cost-efficiencies (Lützhöft et al. 2011). Maritime shipping is heavily influenced by the global economic, trade and environmental trends and were significantly impacted by the economic downturn in 2020 resulting from the COVID-19 pandemic. According to UNCTAD (2020), the total world fleet consists of 98,140 commercial ships over 100 gross tons (GT). Of these, the number of gas carriers, oil tankers, bulk carriers and container ships grew most rapidly over the year to 2020. Despite the advances in technology, processes, procedures, training and regulations, a total of 193 vessels exceeding 100 GT were lost over the 3 years from 2017, mainly through sinking (62%), grounding (15%), fire/explosion (10%), machinery damage/failure (6%) (SSR 2021: 14). The type of cargo and size of vessel have a big impact on the extent and consequences of an accident at sea. Crude oil alone accounted for around 17–20% of total seaborne goods loaded between 2010 and 2019, and the amount of crude oil transported annually averages around 1,800 million metric tons (UNCTAD STAT 2019). In addition to the type of cargo, the increasing size of vessels can impact safety, effective fire prevention and salvage in the event of an accident (SSR 2021), highlighted so vividly by the recent case of the Ever Given ‘wedged’ in the Suez Canal (Guardian 2021).

Over the past 50 years, the size and capacity of vessels have increased by 1,500%, with the largest container ships now being as big as the largest oil tanker and bigger than the largest cruise ships (UNCTAD 2020). According to the ITOPF (2019), between 2010 and 2018, 91% of all oil spills resulted from 10 incidents, an increase from the previous decade where 75% of oil spills resulted from 10 incidents. Indeed, many studies identify collision/allision as a major cause of oil spill accidents in over half of the cases, most occurring while the vessels are underway or in open water (Eliopoulou and Papanikolaou 2007; Uğurlu et al. 2015). The catastrophic and often long-term human, economic and ecological consequences of accidents involving large vessels carrying increased volumes of highly toxic pollutants can be felt globally (ITOPF 2019; Chen et al. 2018). The focus of this study is to investigate human error (HE) in all types of maritime transportation, with a view to better understanding these errors in order to prevent future devastating accidents.

In addition to increasing the size of vessels, another very common way ship-owners reduce their fixed costs is by hiring multinational crews from developing countries or reducing the number of crew members on-board (Lützhöft et al. 2011). This often leads to de-prioritising employee training (Güven-Koçak 2015) and increased communication and comprehension problems between the multi-lingual and multi-cultural crew, who cannot effectively communicate with and understand one another. Crew members also inevitably transfer their cultural perspectives, stereotypes, and racial prejudices, leading to cultural tensions and strained relationships. These tensions are further exacerbated by long working hours, a noisy environment, a sense of isolation and loneliness, poor and often shared living conditions with little privacy, and the impossibility of getting away to enjoy free time alone (Güven-Koçak 2015). Living and working under such conditions for long periods can affect crew morale and raise stress levels, ultimately leading to fatigue, loss of concentration and focus, lower productivity (Alderton et al. 2004) and ultimately accidents.

### Human error (HE) as the central cause of accidents

The complexity and lack of standardisation in maritime accident reporting often mean it is difficult and time-consuming to uncover detailed causal factors (Grech et al. 2002). Despite this, HE has been identified as one of the primary factors in over 75% of maritime accidents (Acejo et al. 2018; Celik and Cebi 2008). In an analysis of 177 maritime accident reports, Grech et al. (2002) found one aspect of HE – lack of situation awareness—to be a severe problem in the maritime domain. Specifically, ‘*shortcomings of the cognitive psychology paradigm of perception, cognition and projection of future events’* (*ibid.* p.2), where HE resulted from a failure to anticipate future actions, a failure to correctly perceive information, a failure to correctly integrate or comprehend information and/or the system. In the context of advancing on-board digital systems, these human failings are particularly concerning as they suggest that as the crew become over-reliant on new technologies, the problems of situational awareness will grow considerably and have more of a negative impact on safety.

*How have researchers considered and framed HE?*

Having reviewed the literature, what is apparent is the different ways in which the concept of ‘human error’ is defined. ‘Human errors’ are the consequences focusing on individual actions leading to errors resulting from intentional actions (Reason 1990), a deviation from the performance of an action (Leveson 2011), a slip (Norman 1981) or a human disturbance that leads to an accident (Rasmussen 2000). For some, HE also includes organisational factors (Reason 1997; Dekker 2006). A selection of these definitions is summarised in Table 1.

**Table 1:** Definitions of human error (HE)

Definition	References
“A slip is a form of human error defined to be the performance of an action that was not what was intended. Slips can often be interpreted. They often appear to result from conflict among several possible actions or thoughts, from intermixing the components of a single action sequence, or from selection of an appropriate act but in some inappropriate way”	Norman (1981, p1)
“The term error can only be applied to intentional actions. It has no meaning in relation to nonintentional behaviour because error types depend critically upon two kinds of failure: the failure of actions to go as intended (slips and lapses) and the failure of intended actions to achieve their desired consequences(mistakes)”	Reason (1990, p15)
'human error' is a judgment made in hindsight"	Woods et al. (1994, p200)
“Human error in complex and potentially hazardous systems therefore involves human action (or inaction) in unforgiving systems”	Kirwan (1994, p3)
“human error is a consequence not a cause, are shaped and provoked by upstream workplace and organizational factors”	Reason (1997, p126)
"a 'human error ' is the post hoc attribution of a cause to an observed outcome, where the cause refers to a human action or performance characteristic"	Hollnagel (1998, p24)
“If a system performs less satisfactorily than it normally does—due to a human act or to a disturbance which could have been counteracted by a reasonable human act—the cause will very likely be identified as a human error”	Rasmussen (2000, p7)
“Human error is systematically connected to features of people tools, tasks, and operating environment. Progress on safety comes from understanding and influencing these connections”	Dekker (2002, p372)
“Human error is usually defined as any deviation from the performance of a specified or prescribed sequence of actions”	Leveson, (2011, p11)

The definition of HE has evolved from being seen as a slip (Norman 1980) to a more complex interaction between people, tools and tasks in an organisational environment (Dekker 2002). How HE is defined is mainly dependent on the perspective of the discipline evaluating it. For instance, from the engineering discipline, HE is considered a set of causes that need to be tackled to avoid accidents. However, from the perspective of human factors and ergonomics (HFE), HE is more complex and includes aspects of organisational factors and has no systematic solutions to solve the causes. However, the terms are generally ill-defined with little distinction between them and are often used interchangeably.

In reviewing human factors that contribute to organisational accidents in shipping, Hetherington et al. (2006) developed a framework highlighting three areas common to accidents that can potentially improve shipping safety if moderated. These are (1) personnel issues (fatigue, stress, health, situation awareness, teamwork, decision-making, communication) which were immediate causes (2) organisational and management issues (safety culture) which were underlying causes, and (3) design issues (automation). As with all such studies, there are acknowledged limitations. In this case, there are only 20 studies, many of which lack measures of the impact of specific human behaviours on accidents. This, however, does not invalidate this study but rather highlights the need for more robust research in this complex area.

Researchers have used techniques such as Human Factor Analysis and Classification System (HFACS) and Fuzzy Analytical Hierarchy Process (FAHP) to further investigate causal links and weightings of HE in shipping accidents. For instance, operator failure due to lack of skills, misperception or error of judgement (Celik and Cebi 2008), fatigue and miscommunication more recently (Ung 2019; Yıldırım et al. 2017). These studies concluded that HE was one of the leading causes of shipping accidents. While these studies offer high level and general insights into the role of shipping, they do not sufficiently explain the role of HE in shipping accidents from an organisational or ecosystem perspective.

By applying a bibliometric review approach, this paper explores the literature in more depth to understand the themes related to the causes of maritime accidents and, more specifically, the aspects attributed to HE.

## Methodology

**Fig. 1:**
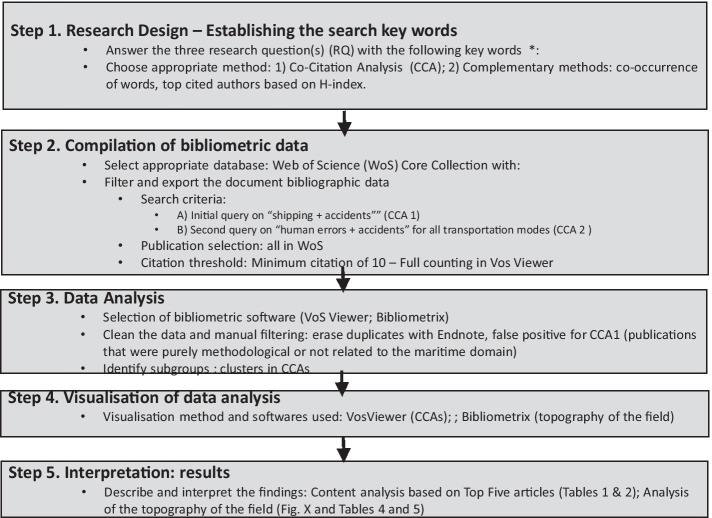
Bibliometric review workflow (adapted from Zupic and Cater (2015:5))

A bibliometric review (BR) methodology was selected for this paper. It is a systematic approach consistent with the paper’s objective of presenting the state-of-the-art of published research on the causes of human error in maritime transportation accidents. Bibliometric reviews mobilise quantitative rather than qualitative techniques, reducing researcher subjectivity and bias, and are increasingly being used by scholars to map the development and structure of a scientific field (Zupic and Cater, 2015). They can combine co-citation analysis and bibliographic coupling to map the network of publications and arrive at distinct clusters of thematically related publications (van Oorschot et al. 2018: 2). Bibliometric reviews also include other complementary analyses such as co-occurrence of keywords (where two or more keywords appear together in a document), co-word analysis (words that occur more frequently together with titles and abstracts) and co-citation of authors (Munim et al. 2020). The bibliometric review in this study followed the workflow process proposed by Zupic and Cater (2015:5) summarised in Fig. 1.

*Research design (Step 1)* The initial broad review of the maritime transportation literature highlighted the important issues of ‘human error’ in maritime ship accidents. Therefore, the research question to direct this study, established at the outset of the paper, is, ‘*what is the current state-of-the-art of the research regarding HE as the main cause in maritime transportation accidents?’* In order to have a complete view of how this human dimension is handled in the field of maritime transportation, the appropriate methods selected were, (1) co-citation analysis to visualise the seminal publications related to these keywords (CCA); (2) the co-occurrence of words to complete the structuring of main topics to provide a topography of the field; and (3) top-cited authors based on the h-index analysis in order to further analyse the most recently developed topics and concepts.

*Compilation of bibliometric data (Step 2)* The Web of Science (WoS), which contains over 33,000 journals, including books, conference proceedings, data sets and patents dating back to 1900, was used as the core database for this bibliometric search. The WOS content is curated by experts and provides the data for Journal Impact Factor scores. The metadata and citation data are considered high quality and reliable (Haraldstad and Christophersen 2015) and, in line with other studies, is considered most appropriate for bibliometric reviews (Zupic and Cater 2015).

The initial search using the keywords “Shipping + Accidents” resulted in 1661 publications and was the basis for Stage 1 of the co-citation analysis. Several false positives were encountered. This is where ostensibly relevant articles that had keywords matching the search terms, on close reading, were found not to be related to the maritime domain and so were excluded. However, the seminal books that were most cited were included in the dataset. Articles that were purely about research methodologies with no relation to the maritime context were also excluded; for instance Yang et al. (2013) focuses only on fuzzy logic techniques and Saaty (1980) focuses only on the Analytic Hierarchy Process (AHP). This resulted in a total of 191 publications. The second search using the keywords “Accidents + Human Error” resulted in 2019 publications and was the basis for Stage 2 of the co-citation analysis (CCA). After filtering the data, this resulted in 225 articles.

For each search, a citation threshold was set at ten, which means that only documents that obtained at least ten local citations were included in the network. Furthermore, the entire counting method was used to select the articles for the CCAs. Any co-authored documents are counted and where ‘a link between [two authors] has a strength of 2 [this] indicates that both authors have co-authored two documents’ (Van Eck and Waltman 2013, p.32).

*Data analysis and visualisation (Step 3 and 4)* To provide a complete bibliometric analysis, we used VosViewer software for the co-citation analysis (CCA) and Bibliometrix software for the bibliometric citation analysis (Munim et al. 2020) to identify the most influential articles, journals, authors and institutions. VosViewer software was used to generate a Co-Citation Analysis (CCA) of cited articles that were co-cited at least 10 times. Regarding CCAs, an overview of the major publications classified in clusters corresponding to seminal themes of interest-based on the dataset collated using the keywords “Shipping + Accidents” (CCA1) and “Accidents and Human Errors” (CCA2) was presented. These are further considered in the discussion section. Bibliometrix software provides a topography of the field with co-occurrence of keywords, a co-word analysis (Figs. 4, 5). Finally, the top 20 authors resulting from the keywords are presented in Table 4 following Munim et al. (2020).

*Interpretation (Step 5)* At this stage, the researchers evaluated the top five papers of each cluster to interpret their content and were labelled according to the keywords (see Tables 2, 3). The analysis of the CCA was supplemented with a topography of the field (analysis of top 20 authors for “Accidents + shipping” and “Accidents + Human error”) and discussed in the following section.

**Table 2:** References output indicators and citation impact related to stage 1 and Fig. 2

Cluster	Cluster label	No. of articles	Top 5 most cited articles*	Average citation (based on top 10 articles in each cluster)
A (Red)	Analysis of human and organisational factors in shipping accidents	73	Reason (1990), Soares and Teixeira (2001), Hetherington et al. (2006), Trucco et al. (2008) and Chauvin et al. (2013)	31.8
B (Green)	Structural and engineering designs	54	Minorsky (1959), Simonsen (1997), Terndrup Pedersen and Zhang (1998), Wang et al. (2002) and Pedersen (2010)	22.3
C (Blue)	Causes and analysis of collisions/ grounding accidents	47	Fowler and Sørgård (2000), Kujala et al. (2009), Montewka et al. (2010), Goerlandt and Kujala (2011) and Li et al. (2012)	30.8
D (Orange)	Risk and probabilistic modelling	17	Montewka et al. (2014a), Goerlandt and Montewka (2015a), Goerlandt and Montewka (2015b), Kum and Sahin (2015) and Banda et al. (2015)	21.6

*Total citation impact of 191 articles

**Table 3:** References output indicators and citation impact related to stage 2 and Fig. 3

Cluster	Cluster label	No. of articles	Top 5 most cited articles*	Average citation (based on top 10 articles in each cluster)
Cluster 1 (Red)	Task analysis and cognitive approaches to improve human reliability	51	Swain and Guttmann (1983), Kirwan (1994), Hollnagel (1998), Shorrock and Kirwan (2002) and Chang and Mosleh (2007)	18.7
Cluster 2 (Green)	Theories and concepts to better understand human error	51	Norman (1981), Rasmussen (1983), Reason (1990), Endsley (1995) and Reason (2000)	23.07
Cluster 3 (Blue)	Human Factor Analysis and Classification System (HFACS) to improve safety	48	Hetherington et al. (2006), Trucco et al. (2008), Celik and Cebi (2008), Chauvin et al. (2013) and Chen et al. (2013)	15.02
Cluster 4 (Orange)	Normal Accident Theory (NAT) vs HRO (High-Reliability Organisations) to explain the cause of the accident	41	Rasmussen (1997), Perrow (1999), Dekker (2006), Leveson (2011) and Hollnagel (2016)	20.39
Cluster 5 (Purple)	Classification of accidents in several industries due to human error	34	Shappell and Wiegmann (1997), Reinach and Viale (2006), ElBardissi et al. (2007), Patterson and Shappell (2010) and Lenné et al. (2012)	49.88

*Total citation impact of 225 articles

## Discussion of findings

### Co-citation analysis (CCA 1): understanding shipping accidents

**Fig. 2:**
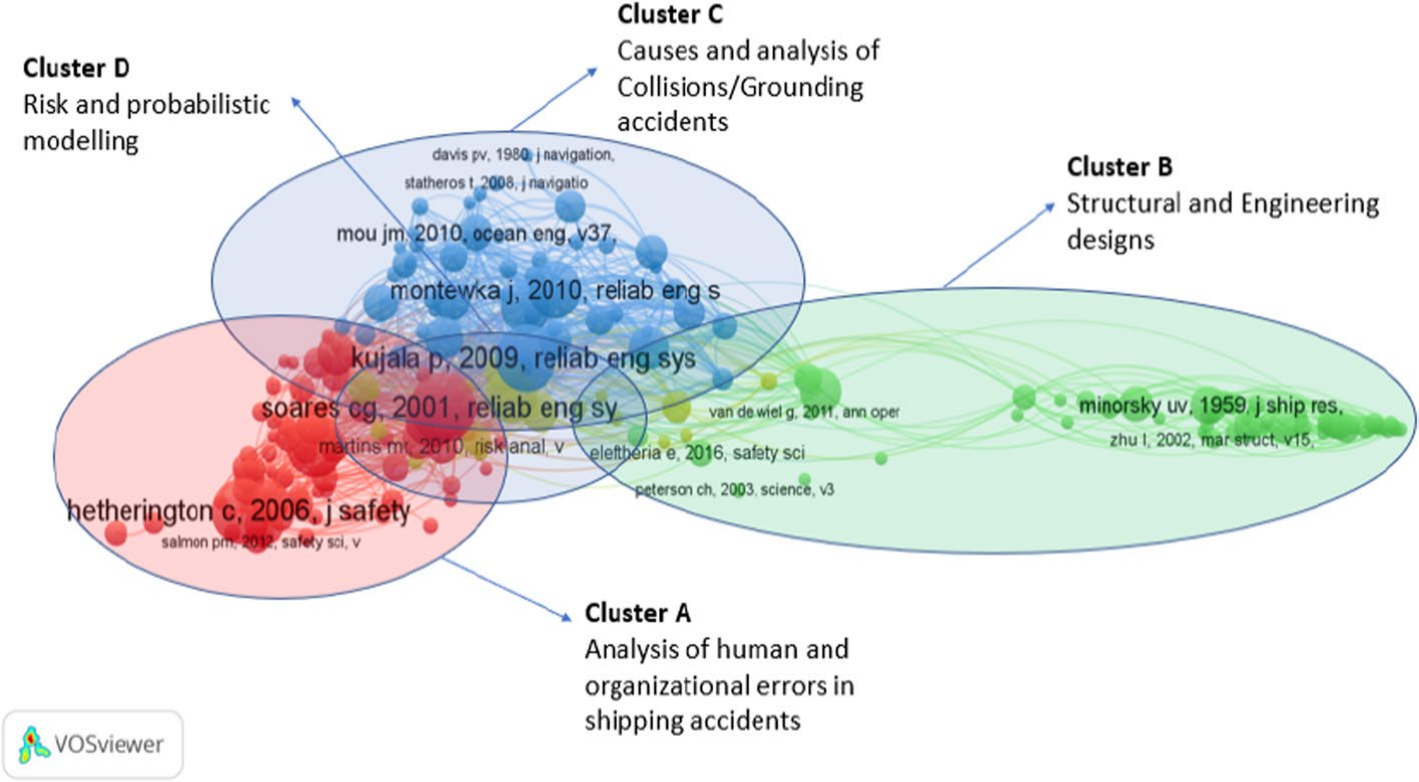
CCA clusters based on keywords “Shipping + Accidents”

The initial query using the keywords “Shipping + Accidents” was grouped into four clusters illustrated in Fig. 2. Two clusters (A and C) focus on human error, whereas the other two (B and D) refer to engineering or other causes.

Figure 2 presents the four main clusters identified in Stage 1 of the bibliometric review with CCA based on shipping accidents and illustrates the clusters with the most weight within the overall map based on the total articles per cluster and the average number of citations per article as summarised in Table 2. Cluster A and C in Fig. 2 focus on finding and/or explaining the causes (with methods such as Root Cause Analysis) of HE. Cluster B deals with technical, engineering and other structural design issues, while Cluster D is related to risk and probabilistic modelling with mathematical models. As Clusters B and D were not related to HE, they are excluded from the analysis below.

- Cluster A is labelled “*Analysis of Human and organisational errors in shipping accidents*”. It gathers 73 of the most cited co-cited references. Most research papers in this cluster describe and/or analyse the human error. In Table 6 (in the “Appendix”), the main themes were classified into three categories: Managerial and Human Resources, Socio-technical use and Individual and Cognitive approaches to explain, predict and/or prevent maritime shipping accidents. Cluster A contains the most significant proportion of references and overlaps extensively with Cluster C (Collision/Grounding accidents).
- Cluster C is labelled *“Collisions/Grounding accidents”*. This cluster has 47 cited references and has extensive connections with Cluster A, which incorporates human and organisational errors as the leading causes of groundings and collisions. However, in Cluster D, HE is considered one of many other mathematical variables in risk models and algorithms; nevertheless, it overlooks the different dimensions that constitute HE (such as fatigue, organisation choices etc.).

This initial search confirms that HE is the central concern related to shipping accidents, highlighted in more than 63% of articles in Clusters A and C. To examine further the results of clusters A and C, all the articles were reviewed by the researchers concentrating on their titles and abstracts. These were classified into three main topics related to HE, namely (1) managerial and human resources, (2) socio-technical use, and (3) individual errors analysed with a cognitive approach. These categories are used to evaluate the literature identified in each of the respective clusters (A and C) and are summarised in Tables 6 and 7 in the “Appendix”.

### Co-citation analysis (CCA2): understanding the role of “human error” in maritime accidents

**Fig. 3:**
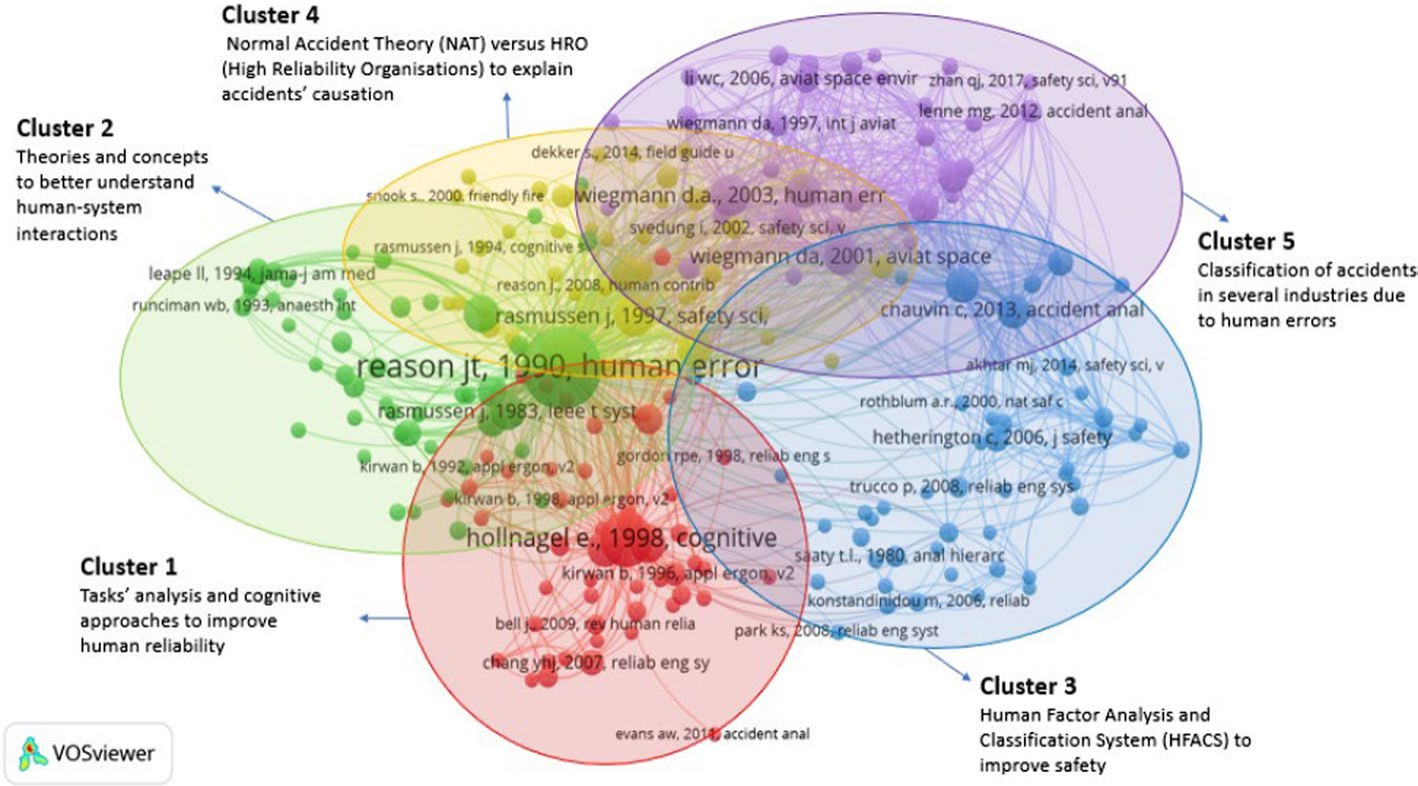
CCA clusters based on keywords “Accidents + Human error”

In the second stage of the CCA process, another query using the keywords “Accidents + Human error” was conducted to refine our understanding of human error. A total of 225 articles resulted and were grouped into 5 clusters described in Table 3 and illustrated in Fig. 3.

Cluster 1 focuses on an individual unit of analysis looking into tasks and cognitive reactions. Cluster 2 proposes the main theories around man–machine interactions (particularly Information Technologies and Systems) with the work of Reason (1990) linking all the other clusters. Cluster 4 adopts a more structural unit of analysis based on ship structures and illustrates the theoretical debate between Normal Accident Theory (NAT) and High-Reliability Organisations (HRO). Cluster 3 is centred in the Human Factor Analysis and Classification System (HFACS) related to safety, and Cluster 5 centres on identifying contributing factors and classifying accidents in several industries.

- Cluster 1 was labelled *Task analysis and cognitive approaches to improving human reliability*. It gathers 51 co-cited references. Most papers propose methods or models to assess the risk of accidents to better predict them (Hollnagel 1998; Swain and Guttmann 1983; Shorrock and Kirwan 2002). Most research approaches adopt a cognitive understanding of HE (Hollnagel 1998; Shorrock and Kirwan 2002; Chang and Mosleh 2007). Kirwan (1994) focuses on tasks performed by humans as they interact with systems or technologies and the related risks. The top ten articles of this cluster are oriented toward improving human reliability.
- Cluster 2 was labelled *Theories and concepts to better understand human-system interactions*. This has 51 co-cited references that are primarily dominated by the work of Reason, who proposed the theoretical integration of several previously independent literatures (Reason 1990). He further proposed two ways of modelling HE: using a person or a systems approach (Reason 2000). Other articles focus on socio-technical use, such as Rasmussen (1983), who develops theoretical backgrounds related to introducing information technology, digital computers and knowledge. Endsley (1995) discusses several methods to measure situation awareness, and Norman (1981) suggests a theory of action to avoid action slips.
- Cluster 3 was labelled *Human Factor Analysis and Classification System (HFACS) to improve safety.* Cluster 3 gathers 48 co-cited references. Most papers investigate human error using the HFACS method to analyse multiple accidents (Celik and Cebi 2008; Chauvin et al. 2013; Chen et al. 2013). Chen et al. (2013) develop an HFACS dedicated to Maritime Accidents. Hetherington et al. (2006) raise the issue of aggregating the causal factors of HE within the maritime context, while Trucco et al. (2008) propose an innovative approach to integrate the human and organisational factors into risk analysis.
- Cluster 4 was labelled *Explaining accident causes using two theoretical approaches*. Cluster 4 gathers 41 co-cited references. This cluster illustrates the theoretical debate between Normal Accident Theory (NAT) and High-Reliability Organisations (HRO) to explain the causes of accidents.
- Cluster 5 was labelled *Classification of accidents in several industries due to human error*. Cluster 5 gathers 34 co-cited references with several classifications of accidents due to HE. Shappell and Wiegmann (1997) propose a taxonomy of unsafe operations. Reinach and Viale (2006) investigate six accidents, highlighting 36 probable contributing factors. Based on an analysis of 508 mining accidents, Patterson and Shappell (2010) classify main causations between operator error and system deficiencies

Overall, Fig. 3 identifies relevant literature tackling managerial and human resources issues. Clusters 3 and 5 adopt quantitative methods and provide statistics and factor weightings to describe the cause of accidents. Cluster 1 represents individual and cognitive issues with HFACS as the main method. Socio-technical issues are addressed in Clusters 2 and 4 but mainly with theoretical approaches coming from psychology, cognitive sciences and ergonomics.

Both CCA1 and CCA2 are complementary. Figure 2 of CCA1 (‘Shipping + Accidents’) provides the whole landscape of seminal publications (including papers and books) related to accidents in maritime transportation. Two clusters of CCA2 are more related to understanding human error in accidents (A and C). To go further, Fig. 3 with CCA2 ‘Accidents + Human error’ provides the seminal books and papers related to the analysis of what human error is, their causes and recommendations to cope with them, whatever the types of transportation. CCA1 is focused on maritime transportation, whereas CCA2 includes all types of transportation modes that tackle the HE question.

There is minimal overlap between CCA1 and CCA2. There are only two authors that belong both to CCA1 and CCA2. One is Reason for his seminal book on HE that is both co-cited in maritime and other transportation fields to study accidents. The other is Hetherington et al. (2006), who is one of the main cited papers to study HE in maritime.

This review highlights the limited understanding of HE and the lack of depth that would fully explain HE and on-board group behaviours, both from human resources and socio-technical perspectives.

## Topography of the research field

To further develop the bibliometric review and compliment the co-citation analysis, the following section presents a topography of the field, following Munim et al.’s (2020) approach, which further maps the structure of the research themes related to the research keywords.

### Topography of ‘shipping and accidents’ research

**Fig. 4:**
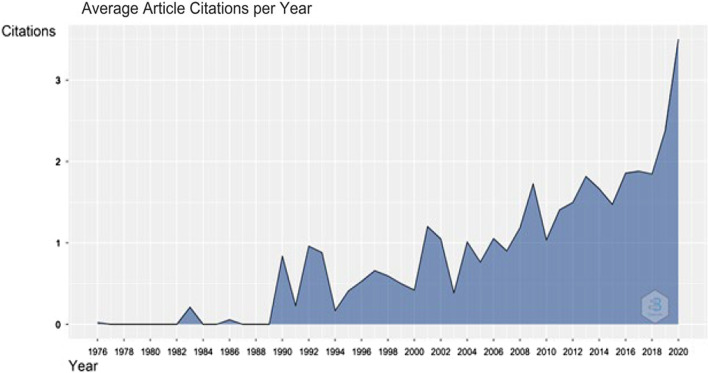
Average citations per year for the keywords “Shipping + Accidents”

There has been a growing trend in the number of articles’ citations related to ‘Shipping and Accidents’, particularly over the last decade, illustrated in Fig. 4. This suggests the growing interest and importance of this topic.

**Fig. 5:**
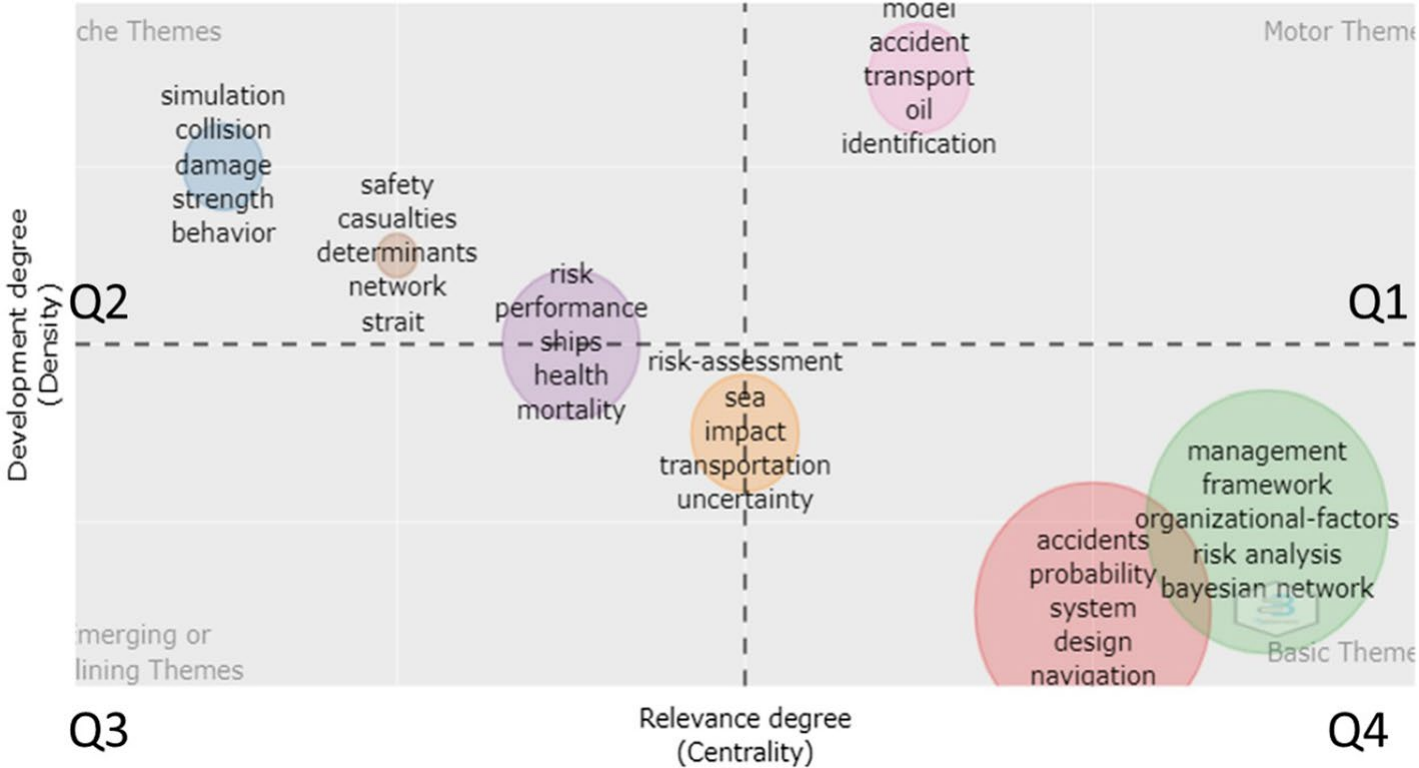
Thematic map with co-occurrence of keywords for ‘Shipping + Accidents’

To understand this trend in more depth, centrality and density measures of the main topics are calculated and presented visually in Fig. 5. Centrality (Callon centrality) measures the strength of association between the keywords in one cluster with another cluster. Density (Callon density) measures the aggregate strength of the relationships between the keywords in the same cluster (Cobo et al., 2011).

Based on keyword co-occurrence centrality, the themes in quadrant Q1 (top right) called motor themes are topics that act as a bridge between other topics. The keywords in quadrant Q2 (top left) indicate highly developed or niche themes. The keywords in quadrant Q3 (bottom left) display emerging topics in a particular field. Finally, the keywords in quadrant Q4 (bottom right) indicate basic and transversal themes currently under development.

This thematic map shows that the most well developed and highly researched themes are related to models of accidents related to transportation, specifically in the context of oil spills and using identification systems such as AIS. In addition, the basic topics that are underdeveloped are related to frameworks, organisational factors, risk analysis and Bayesian networks, followed by probability of accidents related to design engineering.

**Fig. 6:**
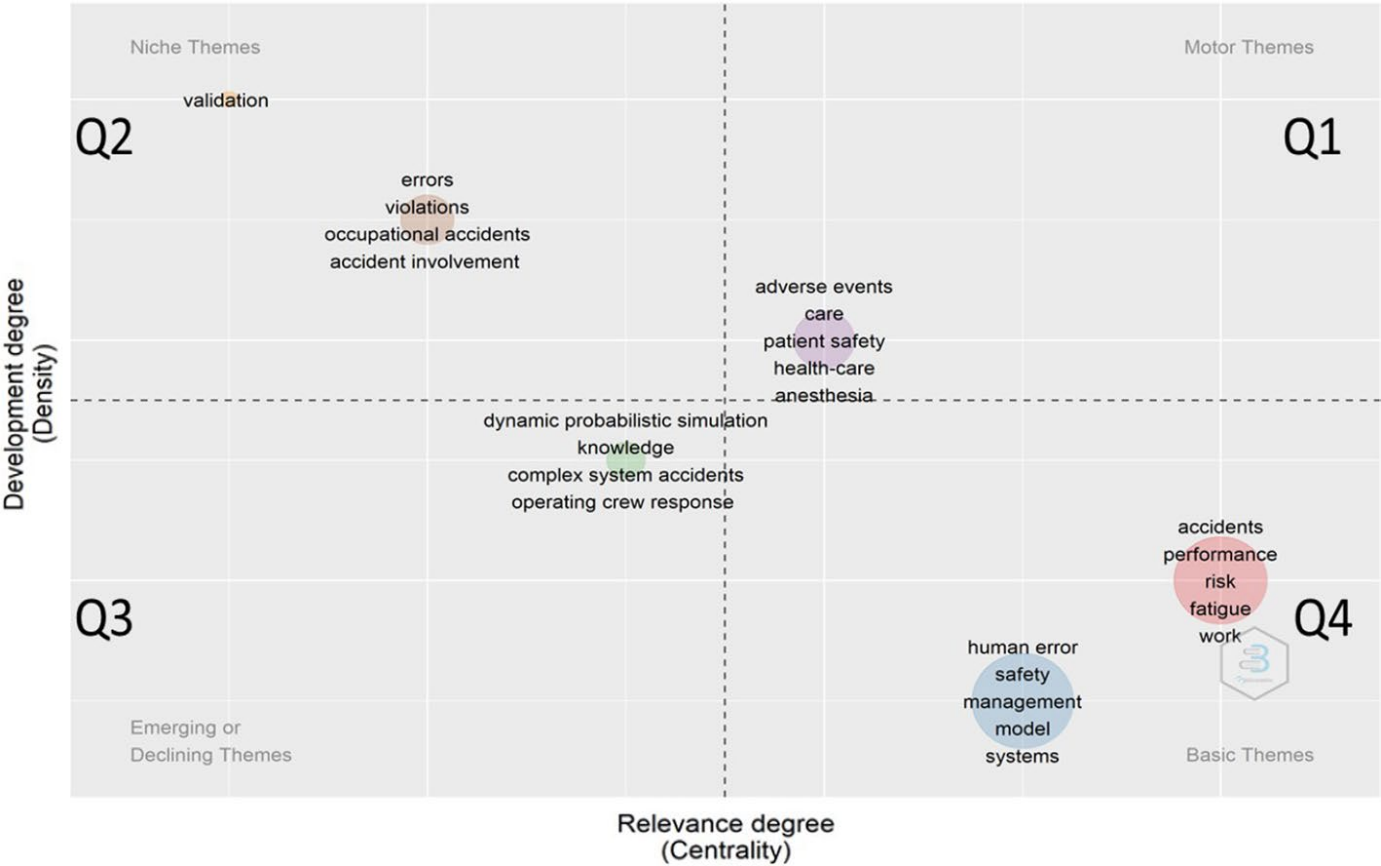
Thematic map with co-occurrence of keywords for “Human error + Accidents”

The themes in the top right Q1 are fundamental to structure the research field. The keywords in the theme (model, accident, transport, oil, identification) are related to accident modelling, oil transportation and identification of risks. Q1 has strong connections with the keywords (sea, impact, transportation, uncertainty) (between Q2 and Q3). The cluster in Q1 is also connected with the themes of Q3 and Q4 regarding quantitative analysis of accidents and behavioural factors. The keywords in Q2 (simulation, damage, collision, strength) are research fields related to collision simulations and strength behaviour simulations of maritime structures. The other cluster (safety, casualties, determinants, network) has specialised themes; it is pretty isolated with strong internal ties but weak relations with other themes. The themes in Q4 (accident, probability, system, design, navigation) are related to a quantitative analysis of accidents, human error quantification and decision making (consistent with clusters B and D of CCA1). The other themes in Q4 (management, framework, organizational factors, risk analysis, Bayesian networks) are related to human and organizational factors related to the maritime industry and risk analysis using Bayesian networks (consistent with cluster C of CCA1). These themes have strong external ties with all other clusters (Fig. 6).

### Topography of ‘human error + accidents’ research

In order to understand shipping accidents in more depth, the topography of the research related to the keywords ‘human error + accidents’ was also developed. While there were studies related to human error in other fields (such as medicine), only those related to the maritime sector are commented upon in this section.

The keywords in the Q4 are basic themes still in development with many external links but not necessarily strong with all the other clusters of Fig. 6. On one side, the cluster related to (Accidents, performance, risk, fatigue, work) corresponds to the themes developed by the top 20 authors in marine technology and reliability engineering (See Table 4). On the other hand, the other cluster with keywords (human error, safety, management, models) is related to the themes developed by the top 20 authors in human factors and ergonomics (See Table 5). The clusters on the Q2 (errors, violations, occupational accidents, accidental involvement) are specialised and isolated themes.

As a conclusion, we can see that the keywords related to the understanding of human error with organisational insights (human error, safety, management systems, organisational factors, accidents, Bayesian network, performance, risks, fatigue and work) are promising fields of research as shown in Figs. 5 and 6. Having highlighted the most interesting keywords and their co-occurrences, we further develop the literature by looking at the top-cited papers of the top 20 authors.

### Focused literature review: shipping + accidents

To review the literature for both keywords “Shipping + Accidents” and then “Human error + Accidents”, the most cited papers of the top 20 authors highlighted by the Bibliometrix software (Table 4) were selected and analysed. Firstly, papers published before 2015 that were cited at least 40 times were selected; second, from 2015 to date (2021), the papers cited 15 or more times were included as they would highlight important and emerging topics. For the keywords “Shipping + Accidents”, this led to a comprehensive database of 222 articles. Table 4 below shows the top 20 authors according to their h-index provided by Bibliometrix.

**Table 4:** Top 20 Authors for the keyword “Shipping + Accidents” based on h-index

Name of top 20 authors	h_index	Total citations	Discipline
Kujala, P	21	1651	Marine technology
Montewka, J	18	1076	Evaluation of risk and safety of maritime transportation
Goerlandt, F	17	1250	Maritime Risk and Safety
Soares, C. G	14	816	Marine Technology
Yan, X. P	14	806	Intelligent transport systems
Paik, J. K	12	283	Naval architecture Marine Engineering
Wang, J	11	760	Marine Technology
Celik, M	10	471	Marine Technology
Akyuz, E	9	278	Maritime Transportation and Management Engineering
Khan, F	9	340	Safety and risk engineering
Zhang, D	9	486	Intelligent Transport Systems
Abbassi, R	8	234	Safety and Risk Engineering, Environmental Modelling
Amdahl, J	8	251	Marine Technology Structures
Weng, J. X	8	200	Road and marine traffic Safety
Garaniya, V	7	226	Environment, Energy and Safety Engineering
Hanninen, M	7	552	Bayesian network
Hansen, H. L	7	300	Electrical & Computer Engineering, Speech Processing Language Technology
Wang, Y	7	273	Intelligent Transport System
Yang, Z. L	7	306	Modelling of safety, resilience and sustainability of transport networks
Zhang, J. F	7	295	Intelligent Transport Systems

All papers most cited and more recently published enter into one of the cluster labels of CCA1. Below we only focus on papers related to clusters A and C as they are related to HE in shipping accidents. Papers related to Cluster A split into two categories: first, some papers focus on the scope of HE from an individual cognitive approach with several methods: second, other papers adopt a monograph or historical approach to highlight human factors.

Firstly, Celik and Cebi (2008) develop a Human Factor Analysis and Classification System (HFACS) for HE in shipping accidents to improve group decision-making. In this model, the organisational influences are described as “big categories” (resource management, organisational climate and organisational processes). Supervision causes are described as inadequate or inappropriate. Finally, “communication, coordination and planning factors” are categorised as “personnel factors” and considered group-related activities. These models provide useful categories but do not fully describe how organisations act in a dynamic context.

Secondly, Graziano et al. (2016) propose a classification of HE taxonomy based on collision and grounding reports with four main categories: task errors, cognitive domain, technical equipment and performance. Interestingly, internal and external communication errors are highlighted as one key task; external communication includes communication between pilots, other vessels, tugs, VTS and on-shore. The main novelty of this paper is the description of the leading technical equipment which mediates HE, the most frequent being radars, followed by VHF and paper charts. All in all, we can infer from these categories of errors that they occur in situations where internal teams and/or external groups and stakeholders are involved.

Thirdly, Wu et al. (2017) propose a cognitive reliability and error analysis with evidential-based reasoning with original variables such as linguistic issues and incomplete information on-board. Akyuz and Celik (2014) similarly provide an HFCAS model combined with cognitive maps and highlight, in all categories of the model, the lack of knowledge or training as the major causes of accidents. They recommend studying ships in team contexts (including better diversity management on-board) and training them to adapt according to unexpected circumstances. In this paper, the recommendations are drawn on the necessity to adopt continuous learning, whatever the categories of HE.

Regarding monograph or historical approaches, Islam et al. (2017) develop a monograph for HE in operations maintenance useful for chief engineers and captains. Interestingly, the major causes of accidents come from deficiencies in knowledge (lack of experience) or insufficient training followed by seafarers' fatigue. Hansen developed several historical analyses of death on-board that enlarged and refined the human factors currently considered in studies. For instance, in their analysis, which covers the period between 1986 and 1993, Hansen and Pedersen (1996) concluded that the maritime workplace is a high risk where half of the deaths are due to the workplace and the lifestyle of seafarers. Hansen and Jensen (1998) undertook a unique study on the risks related to female seafarers and showed that major risks are due to their lifestyle (notably the consumption of alcohol and tobacco) and the fact that they “adopt the traditional male jobs at sea”. Roberts and Hansen (2002) highlighted several factors that concern both individuals (notably the age of the vessel as being one of the most important ones), several factors related to the working conditions (such as change of ship due to lost employment, daily routine duties, lifestyle) and the use of space on board (walking from one place to another, falling in docks when hazardous access and working practices are adopted). In a nutshell, most results of this cluster are oriented toward results aiming at facilitating decision-making but mostly at the level of individuals.

Complementary papers related to Cluster C are characterised by a diversity of research methods such as Bayesian networks (Hänninen and Kujala 2012), identification of events and processes of risks (Montewka et al. 2014b), what-if analysis, association rules (Weng and Li 2019), scenario-event tree (Chai et al. 2017), binary logistic regressions (Weng and Yang 2015) and accident reports (Wróbel et al. 2017). They also sometimes develop research on specific ships such as ROPAX, cruise ships or tankers. Finally, they also propose tools or methods that improve safety: for instance, a ship collision alert system (Goerlandt et al. 2015) or a method for detecting possible near-miss ship collisions (Zhang et al. 2016).

This cluster provides interesting categorisations of human and organisational factors but always in “big categories” regarding the organisation of ships that remain static (except for Aps et al. 2015) and still mainly focused on “individuals” as units of analysis and not groups or networks. For instance, Hänninen and Kujala (2012) highlight the changing course in an encounter situation, the officer of the watch, the situation assessment, danger detection, personal conditions and other distractions (maintenance routines, fatigue, bridge view) as the main causes of accidents. Hänninen and Kujala (2014) integrate a new and interesting variable—the role of port state control in accidents—broadening the scope of study from the ship to her wider network. Regarding the automation and digitalisation of ships, Wróbel et al. (2017) provide one of the few analyses of the evolution of accidents with unmanned ships, arguing that if the number of navigational accidents falls, other types of accidents, such as fire on board, will increase with potentially worse consequences.

All in all, these pieces of research provide an interesting categorisation of the causes of HE. However, they all remain static pictures without providing a dynamic analysis, which would be a good basis for adaptive decision-making in specific contexts and building learning recommendations. Most studies still focus on individuals as units of analysis; few consider groups, and even fewer include the whole network of the ship. Research that includes “organisational factors” does not describe their workplaces nor the working conditions and routines on-board. Few studies recommend the necessity of a dynamic learning culture on-board offering ships the possibility to continuously adapt to the unexpected. This paper contends that these approaches will provide an in-depth understanding of the causes of accidents on ships, moving from a “technical structure” described through static categories to a real organisation with human beings on-board, able to adapt accordingly to their specific contexts. Finally, even though the digitalisation of ships is a reality, very few studies consider the use of technical tools as a cause of potential accidents.

### Focused literature review: “human error + accidents”

The search keywords used led to papers related predominantly to transportation modes in aviation or rail. However, all papers related to maritime transportation that were cited 10 or more times were all included (see Table 5). The papers were reviewed to ensure a complete understanding of the content and themes within them. This led to a complete database of 241 articles.

**Table 5:** Top 20 Authors for the keyword “Human error + Accidents” based on h-index

Name of top 20 authors	h_index	Total citations	Discipline
Stanton, N. A	15	703	Ergonomics and Human Factors
Salmon, P. M	14	778	Human Factors and Sociotechnical Systems
Akyuz, E	9	293	Marine Transportation Engineering
Walker, G. H	9	300	Human Factors
Wang, J	9	644	Marine Technology
Celik, M	8	423	Marine Engineering
Khan, F	8	260	Safety and risk engineering
Mosleh, A	8	475	Reliability engineering
Seong, P. H	8	109	Nuclear instrumentation control and human factor engineering
Shappell, S. A	8	606	Human Factors
Wiegmann, D. A	8	646	Human Factors and Systems Safety
Abbassi, R	7	230	Safety and Risk Engineering, Environmental Modelling
Dekker, S. W. A	7	297	Human factors and safety
Jung, W	7	91	Human Factors
Lenne, M. G	7	312	Human Factors
Nazir, S	7	93	Human Factors
Waterson, P	7	150	Human Factors and Complex Systems
Garaniya, V	6	195	Environment, Energy and Safety Engineering
Kim, J	6	82	Human Factors
Rizzo, M	6	405	Biological psychology and cognitive science

The close analysis of the top 20 authors revealed three main academic disciplines that are currently structuring the field grouped as follows: (1) Human Factors and Ergonomics (HFE) on one side and (2) Marine Technology and Transportation Engineering (MTTE) and Reliability Engineering (RE) on the other side. HFE constitutes 11 top-cited authors^2^ and publishes topics inspired from clusters 1, 2, 3 and 4 in CCA 1; (3) MTTE and RE consist of six authors^3^ who publish on topics related to those in Clusters 3 and 5. The research of these authors is in the context of different modes of transportation (including maritime, rail, road, aviation) or other industries (health, mining, nuclear). Some authors are specialised in specific transportation modes—for instance, Shappell and Wiegmann in aviation, Mosleh in nuclear and Akyuz and Celik in maritime.

Our analysis highlights two main contributions of HFE:

1. Frameworks or models based on complex systems and sociotechnical systems theories (such as ACCIMAP, Human Factor Analysis and Classification System (HFACS), Systems Theoretic Accident Model and Processes (STAMP), Causal Analysis based on STAMP (CAST), Critical Path Analysis EAST, Functional Resonance Analysis Method (FRAM) to better assess risks based on taxonomies of human errors. Jenkins et al. (2017) and Hulme et al. (2019) propose a good synthesis and comparisons of them.
2. A diversity of industries and transportation modes can benefit or complement others (Banks et al. 2019; Grant et al. 2018; Hulme et al. 2019). There is, for instance, a historic move in the literature from research in the aviation industry that started to study the concept of situation awareness that is then applied to the maritime context. Indeed, Grant et al. (2018) recently proposed a generic accident causation model that could fit several industries using ‘systems thinking’.

There remain gaps and limitations in the HFE literature. For instance, the term HE does not sit easily with sociotechnical systems theories and concepts on which all these frameworks and models are based (Stanton et al. 2016), and specific phenomena such as the effects of communication and compounded information on performance are still under researched. Another limitation is the difficulty to model the different flows of information between separate teams (Jenkins et al. 2010). Furthermore, except for Harvey and Stanton (2014), there is still very little research focusing on the cognition of systems and large and distributed networks as units of analysis. An exception is Salmon et al. (2015) who study situation awareness at the level of systems. They present ten challenges for improving the understanding of interactions between social, technical and organisations, integrating the openness in systems, developing an understanding of what happens across boundaries (notably communication and coordination), culture, responsibility (with external pressure) and finally emerging behaviours (being more adaptive) and the ability to cope with changes. All these are still relevant and remain potentially fruitful areas for future research.

In the area of MTTE and RE overall, researchers tend to quantify HE in order to avoid researcher subjectivity using a range of methods such as fuzzy process on HFCAS (Celik and Cebi 2008), methods to set up the probabilities of human errors with the Error Producing Conditions (EPC) (Akyuz and Celik 2016) or weights related to causes (Akyuz et al. 2017) or the development of human error indexes (Khan et al. 2006). These methods are sometimes complemented by qualitative approaches such as the Why-because graphs of Chen et al. (2013). Furthermore, research in this field examines accidents in fine-grain looking at the specificities of different types of accidents, such as grounding (Akyuz and Celik 2015), fire (Akyuz et al., 2018), explosions (Baalisampang et al., 2018), offshore (Khan et al. 2016; Ren et al. 2008; Islam et al. 2017), and also different types of ships (Akyuz et al., 2017). To a lesser extent, there is also some research into the interactions between human and information systems (Mokhtari 2007).

However, similar to HFE, there are also gaps and limitations in the MTTE and RE literature that can provide an opportunity for future research. For example, much of the literature in this field, that highlighted that most current causes of HE relate to collective actions, is based on the modelling and analysis of cognitive and individual units of analysis (for instance, Akyuz and Celik 2014), which are mostly related to stress, fatigue, health except for Fan et al. (2018); Fan et al. (2018) mention the emotions of seafarers. Moreover, while Baalisampang et al. (2018) extended these individual factors to include elements such as knowledge, competencies, expectations, goals and attention, combined with workplaces factors (site and equipment design, work environment) and managerial factors (organisation of work, job design and information transfer), these are still not fully developed. Furthermore, when reviewing accident reports (for instance, Baalisampang et al. 2018), researchers do not address the lack of standardisation of these reports (Celik and Cebi 2008), which is a considerable limitation and an area for future work. Finally, as ships are becoming increasingly more automated, there are still very few studies investigating the on-board use of information systems and technologies and their interactions with the shore to improve communication and coordination.

All in all, this previous work has built a solid foundation for analysing HE to better prevent accidents. In the research agenda below, we propose how organisation and management sciences can bring new insights to advance human error research in maritime transportation.

## Research agenda: propositions for studying human error in maritime accidents

Having evaluated the findings from the bibliometric review, it was clear that accidents are mainly explained from an engineering perspective. Human errors remain under-explored from organisational and network perspectives. In this section, five propositions for theoretically framing future research approaches are presented. Each of these theoretical management approaches can help improve our understanding of HE in the context of maritime accidents.

1. Ships as organisations: a novel perspective

The findings from this study revealed that the literature on maritime accidents has not fully conceptualised ships as organisations. Neither has it considered how these organisations behave according to the different temporalities in navigation. So, apart from individual and cognitive-based approaches, how can ships be conceptualised as organisations? Here, the conceptualisation of ships as temporary organisations generally follows navigational routines but, in cases of imminent accidents, develop crisis navigation routines.

From this perspective, merchant ships can be considered as organisations that go from point A to point B in order to deliver products. They are characterised by an organised (collective) course of action ‘aimed at evoking a non-routine process and/or completing a non-routine product’ (Packendorff 1995). Routines are defined as “repetitive, recognizable patterns of interdependent actions, carried out by multiple actors” (Feldman and Pentland 2003). The temporary time frame of the navigating crew is particularly relevant when considering safety management on-board. This is similar to project-based organisations characterised by a once-in-a-lifetime task with a predetermined delivery date, subject to performance goals and consisting of several complex and/or interdependent activities (Packendorff 1995).

Indeed, the analogy of merchant ships and temporary organisations is helpful to distinguish two types of temporalities: regular navigation and the period before an accident. When there are no accidents, the ship’s organisation and the environment are stable most of the time. The objectives of the ship are clear (to go from point A to point B), and actors behave according to a highly centralised and rational organisation that follows relatively standardised and shared routines (Degani and Wiener 1993), which we call ‘regular routine navigation’. This is empirically similar to formal quality management systems. However, during the period just before the accident (which can be short depending on the context), the crew and its network (notably for remote-controlled ships) try to make sense of the situation and adapt to it. Adopting a routine lens to study how routines cease or are transformed during an accident could be an interesting perspective yet not explored.

The transition between ‘regular routine navigation’ and ‘crisis routine navigation’ depends on the type of accident and can range from a few minutes to hours or days. During this transition time, which we term ‘crisis routine navigation’, actors on-board are aware of the imminence of the accident; behaviours on-board change due to uncertainty. As a result, there is an increase in stress (Sheridan 2008) that may lead to phenomena such as “out-of-the-loop” performance. This is characterised by actors’ failure to observe parameter changes and intervene when necessary, an over-reliance and absolute trust in information technology artefacts, a loss of situation awareness and finally, deterioration of an actor’s manual skills (Kaber and Endsley 1997). In such circumstances, both social cooperation modes and decision-making are affected. In the case of disaster management, resilience is critical. This is the system’s ability to anticipate and respond to anomalous circumstances to maintain safe functioning and recover and return to a stable equilibrium (Sheridan 2008; Normandin and Therrien 2016). Further research is needed to study ships as organisations that also include the specificities of their culture.

In the literature, as highlighted in Fig. 3, the leading theory related to ships seen as organisations is the debate between High Reliable Organisations (HRO) and Normal Accident Theory (NAT). This controversy questions two domains, which raises new research questions: firstly, are there alternative theoretical models that can describe ships in practice? Second, with all the technologies and potential resources available today to secure ships, is it still relevant to consider the assumptions of NAT as reliable?

2. Ships: High Reliable Organisations (HRO) or self-organisations embedded in ecosystems?

Arguably, ships can be characterised as HRO and are perceived as one of the most highly centralised and rational types of transportation modes. Like the airline industry, maritime navigation has adopted standardised routines such as Cockpit Resource Management (CRM) implemented to provide checklist procedures that need to be accomplished by coordinated actions and communications between the captain and the other pilot(s) in a flight (Degani and Wiener 1993). According to the ‘high-reliability theory’, extremely safe operations are possible, even with extremely hazardous technologies, if appropriate organisational design and management techniques are followed (Sagan 1993).

However, accidents still do happen in HRO. Normal accident theory (NAT) presents a much more pessimistic prediction – specifically that ‘serious accidents with complex high technology systems are inevitable’ (Sagan 1993:13). This empirical observation presents new research questions, such as, is *the NAT still relevant today? Should we extend HRO theory to propose new concepts that would better describe ships as they function in real conditions? Could another way to manage resources and trade-off decisions concerning investments on ships avoid accidents? Has the maritime industry learnt from the aviation industry (International Air Transport Association congress of 1975) that it is machines that have to be adapted to human-beings and not the reverse *(Clostermann 2017:20)?

By applying Normal Accident Theory, ships can be considered to be an assemblage of components that are self-organised. From this perspective, we propose that ethnographic studies can better describe and shed light on working conditions on ships in real-life settings. From a theoretical perspective, we suggest exploring new concepts to study ships, notably in the case of imminent accidents. For instance, applying the concept of self-organisation of different maritime agents/stakeholders coordinating ports, ships and operations (Caschili and Medda 2012; Watson et al. 2021). More broadly, as ships are being increasingly managed remotely, this implies that their whole ecosystem and interactions with other stakeholders need to be considered in any future research. This includes the near network of shipping (incorporating the ship owner, insurances, port state control, VTS) and in a larger ecosystem representing the choices of the whole industry (flag ship, meta-organisations, countries that develop their marine policy).

Even though ships can be characterised as HRO, the proposition here is that their real organisational mode may be closer to self-organisation depending on the temporality of the accident. This is in direct opposition to the HRO view. The response to any accident is organisationally hierarchical and procedures officially documented according to quality management linked to the International Maritime Organisation (IMO) (Ismael 2011).

3. Digitalisation of ships and management of information systems

Many maritime vessels already use a range of information technologies (IT) and information systems (IS) with a host of different navigational equipment and sensors to assist them to navigate safely and efficiently, including Electronic Chart Display Information System (ECDIS) as a modern replacement for paper-based navigational charts, the Automatic Identification System (AIS) and radar (Radio Detection and Ranging) help improve situational awareness of other vessels and obstacles (Harti-Mokhtari et al., 2007). Furthermore, as Artificial Intelligence (AI) and machine learning develop at a pace, more vessels are using autonomous and semi-autonomous technologies that are monitored remotely from shore-based facilities requiring highly reliable and efficient communication channels (Hogg and Ghosh 2016).

These new technologies and other integrated bridge equipment mean that crew on-board ships increasingly rely on them. “Unlike in static situations where human–machine systems have complete control, in dynamic situations like navigation, changes occur rapidly giving only partial control to the operator” (Hoc 2000: 835). This creates socio-technical systems that incorporate complex interactions between humans, machines and other environmental aspects (Baxter and Sommerville 2011). In this context, three main settings are particularly impacted by the socio-technical use of IT/IS, where human error can occur. Namely, IT/IS implementation, IT/IS use in navigation practice and IT/IS-based decision-making. For instance, the improper consideration of human–computer-interaction in the design of the technologies, the often ad-hoc way in which new and emerging technologies are implemented, and inadequate user training can all lead to inevitable human error (Lützhöft et al. 2011).

Similarly, the objectives of improving navigation safety are inextricably linked to a set of daily decisions taken by several interdependent actors on-board. This process is increasingly dependent on the diffusion and integration of data, information and knowledge between humans and technological devices in order to make decisions and take appropriate actions. Poor systems interfaces and improper allocation of functions to human and computer controllers can result in misinterpretation and misunderstanding of data and information being displayed, which leads to poor decision-making, degraded performance and ultimately accidents (Kaber and Endsley 1997).

Although ship systems are becoming increasingly well-equipped, technologically advanced and more reliable (Rothblum 2002), maritime accidents still happen. No technology is used in isolation, but rather the maritime system incorporates people, the environment (socio-technical and natural), and the organisation. In order to better understand the complexities, issues and problems, and how to avoid the repetition of accidents, all the different IT/IS technologies on-board a vessel must be considered holistically as part of the complex maritime ecosystem (Güven-Koçak, 2015; Watson et al. 2021). This digital transformation in the industry driven by new technologies such as AI and big data generates new operational challenges and risks such as cyber-attacks for the maritime sector that need further investigation (Munim et al. 2020).

One theoretical lens suggested continuing to develop complex systems and sociotechnical systems theories. Ships can be considered complex systems through this theoretical lens, both internally as an organisation and concerning their environment (Sovacool, 2008). These are large, tightly coupled systems (Perrow 1984) where socio-technical interdependencies (Thompson 1967) are high due to their complexity. Internally, a ship is a complex system involving a collection of crew members and the range of instruments and computer networks that support them. None of the crew possesses the complete plan or vision to navigate the ship. However, collectively they use information from the crew in conjunction with instrument observations and procedures to keep the vessel on the course (Ismael 2011). The more complicated the interdependence of systems and subsystems, the higher they become prone to failure due to their complexity, speed of interaction, tight coupling and limitations of their human operators and their designers (Sovacool 2008; Lützhöft et al. 2011: 285). Consequently, from this perspective, ship-related maritime accidents can be characterised by a high level of complexity due to the interrelations of multiple and combined causes and the variability of contexts.

Orlikowski’s (1992) structuration theory, where technology is embedded with structure, can also offer insights into how human agents carry out their routines and the intervention that changes the relationship between human agents and organisational structure (Barley 1986) in the maritime context. Since technology is not always used by knowledgeable agents, this theoretical lens can explain how agents use these new technologies in their daily routines, and how they enact new structures or “technology-in-practice” (Orlikowski, 2000) to better understand human error.

De Vries (2017) is one of the few researchers in the maritime domain that showed how navigation safety of seagoing vessels can be improved through the socio-technical interaction of humans, technology, organisations and the environment drawing on Hollnagel et al.’s (2014) Functional Resonance Analysis Method (FRAM). Building on this work, De Vries and Bligård (2019) further demonstrated the benefits of applying a socio-technical systems perspective to influence navigation assistance assessment and design. Furthermore, they showed how discussions with stakeholders such as users, designers, managers, and regulators contributed to safe operations in the maritime context. However, these studies are few, and by applying a socio-technical perspective to the design of on-board systems to ensure they are compatible with and adapted to the human operator to improve performance (Brett et al. 2011) is a fruitful area of research for understanding and ultimately reducing human error in maritime transportation accidents.

As a consequence of these fast-paced technological developments, further research is needed on the interaction of ships within their broad and complex maritime ecosystems. These include but are not limited to the maritime environment, navigation and technologies, and the international organisations that frame, govern and regulate today’s shipping industry. This idea of improvement relies on developing standards in an industry that is more and more digitalised and interconnected (Watson et al. 2021). By improving our understanding of the maritime industry's emerging needs, which is partly considered self-organisations within an ecosystem, and partly tightly coupled with other systems, future accidents can be reduced.

4. Power Lens: a missing link

Organisations of all types, including ships and their ecosystems, are fundamentally underpinned by power relationships and issues. However, there is limited literature on this topic in the maritime context. At the level of the ship, a unique aspect of maritime culture is absolute autonomy and a strong power culture where the captain, known as “master under God”, is in full charge. While at sea, the captain has full authority over the ship, her occupants, and operations and is responsible for all safety issues (Güven-Koçak 2015), including final decisions and the responsibility related to accidents such as grounding. The captain and officers can exercise their judgement to make necessary decisions, such as changing routes, arrival ports or schedules.

With increasing links between the sea and the shore, communications between the ship-owner, who manages the ships from the shore, and the captain who stays on-board, may sometimes not be very effective. For example, in the Torrey Canyon oil tanker wrecked off the coast of Cornwall, this was initially attributed to several human errors. However, a more detailed examination identified management decisions ‘that put pressure on the captain’ and ‘equipment design issues’ related to activation of the autopilot mode (Harvey et al. 2013) as contributing factors to the disaster. Despite this, the literature hardly mentions in any depth communications issues between the vessels at sea and the shore and the pressure from the shore, in some cases due to trade-offs between security and profit that the captain and its crew experience. The few papers that deal with this issue mention “external pressure” as a factor without providing any details.

At the level of the ecosystem of ships, having multiple actors in this domain makes it difficult to legally assign responsibilities in the case of an accident. Empirical data suggests that diverging political interests stall proper investigation and prevention of similar accidents. Thus, the appearance of a mysterious oil spill on the north-east coast of Brazil in September 2019 is most probably linked to crude oil from Venezuela that was carried by the Greek-flagged ship Bouboulina (BBC 2019). There is strong evidence that the company, the captain and the vessel’s crew failed to communicate to authorities about the oil spill/release of the crude oil in the Atlantic Ocean.

The broad literature on power is diverse and complex, and its ramifications for the study of organisations have remained largely unexplored (Haugaard and Clegg 2012), especially in the maritime transportation sector. Indeed, power concerns the ways that social relationships shape capabilities, decisions and changes within organisations. Organisational power is bounded by the capacity of the decision-makers to gather and analyse complex data, which are often multi-dimensional and constrained by prior experiences, learning and knowledge (Haugaard and Clegg 2012). As such, the sources of power—reward, sanction, expertise, reference value and legitimacy—can also trigger conflict, especially when there is a divergence of objectives and strategies for achieving those objectives (Fulconis and Lissillour, 2021).

Of the few studies that examine power, Lissillour and Bonet Fernandez (2020) adopt a Bourdieusian perspective to understand the balance of power in the governance of the global maritime chain. They highlight the conflict of interest between the different global maritime stakeholders. In the context of human error and accidents, the maritime transportation stakeholders – which includes vessel owners, ship captains, classification authorities, insurers, customers and many others – often have differing and competing priorities between safety and economic interests. Often their strategies for managing these most effectively also diverge, leading to tensions and conflicts and ultimately trickle down to operational and human errors resulting in catastrophic accidents. Research should be developed to further understand the interactions among all the stakeholders at the level of the network of actors cooperating in the case of accidents, including the meta-organisations in this wider network (Berkowitz and Dumez 2016) acting to regulate the industry and sustain the oceans.

In the context of maritime transportation, there are several meta-organisations (Berkowitz and Dumez 2016) operating to regulate the industry with significant consequences on the collective actions of ships in their daily activities. More research is needed to build on Harvey et al. (2013)'s work to further develop and mobilise the concept of meta-organisations. Other theoretical backgrounds, such as neo-institutionalism (DiMaggio and Powell, 1983), can shed light on potential isomorphism behaviours at the industry level. This can then be applied to the maritime context to explore how to better cope with accidents, reduce their often catastrophic consequences, and ultimately reduce them.

Since organisations are neither rational nor natural, the theories of power can translate practice to theory and highlight the phenomena of changing organisational practises (Haugaard and Clegg 2012). Thus, future studies could use the lens of power theories with human error in the maritime accident context at the centre of the analysis to better understand communication and coordination issues and the stakes and conflicts of interest of the power relationships between the different actors.

5. Developing dynamic safety capabilities for learning ships

In addition to more collaborative relationships, each ship and related stakeholders should develop their capacities to learn from the past to reduce future accidents. In this area, we propose to develop the concept of dynamic safety capability within the literature on learning organisations. Several streams of research have explored how organisations can learn from rare events such as crises or accidents. Developing alertness to weak cues in the environment is the first step for developing intelligence. Attentional triangulation (Rerup, 2009) combines three forms of attention – stability, coherence and vividness- for anticipating and preventing unexpected events. Previous studies have tended to base their analysis on the concept of situation awareness, mainly focusing on individuals (Hetherington et al. 2006). Very few studies have mobilised situation awareness through teams and systems (Stanton et al. 2015). Thus, dynamic capabilities can provide an interesting perspective for encompassing the previous concepts concerned with issues of adaptation and growth.

Different kinds of dynamic capabilities have already been identified in the literature. A dynamic safety capability is an organisation’s capacity to “generate, reconfigure, and adapt organisational routines to sustain high levels of safety performance in organisations characterised by change and uncertainty” (Griffin et al. 2016: 249). Dynamic safety capability relies on three processes of organisational learning. Experience is first accumulated through tacit learning from ongoing action and events. Then the tacit learning is articulated and shared through collective discussions and processes of sense-making. Finally, knowledge is formalised into regulatory procedures (Griffin et al. 2016). Since crises remain rare events, the authors suggest using the simulation of high-risk environments and their potential consequences to allowing participants to engage in sense-making and focus on team communication and coordination processes. This literature provides rich insights into the importance of developing the ability to share knowledge and learn. However, most of the disaster cases investigated by Griffin et al. (2016) dealt with stable organisations.

Further research could focus on the mechanisms, processes and related skills for developing a safety capability aboard extreme cases such as tankers. In such temporary organisations, a salient issue is the ability to share knowledge among highly dispersed teams in terms of role tasks. In addition, these teams that frequently change, have to manage the continuity of routines through periods of transitions. These organisations, partly similar to SMEs, have to develop a certain level of absorptive capacity (Benhayoun et al. 2020) to identify and capture the external information that comes from the ecosystem to support on-board decision-making. The temporality of ships, which partly prevents routines for learning from rare events, questions how they can become learning organisations. This raises new research questions such as: How could we reconcile ships being both temporary and learning organisations? What is the subculture that would allow ships to move from a culture of adjustment (Baumler et al. 2020) to become learning organisations?

## Conclusion

Under the umbrella term of “human error”, the literature presents many different explanations for accidents, including flaws in structural and engineering designs, cognitive limits and organisational choices. Can all these causes be considered to be “human” errors? In principle, at some point, the causes of all accidents can be related to the “human”, but in providing such a vague catch-all term, the real issues fail to be identified and addressed. This paper suggests that research from the disciplines of human and social sciences, particularly organisation studies, can provide new and relevant insights by clarifying how ships can be described in terms of organisations and by considering them in a whole ecosystem and industry.

The main contributions of this paper are twofold. First, four thematic clusters were identified through a bibliometric review of the causes of maritime accidents related to human error. Among them, the analysis of human and organisational errors showed that the three main causes are related to human resources and management, socio-technical IT/IS, and individual and cognitive errors. A second search on “human error” highlighted five clusters that confirm these three main root causes and provide several references for each of them. Second, the paper provides a critical analysis of the papers published by the top 20 authors cited both for shipping accidents and human error. Finally, several theoretical concepts and propositions for future researchers and practitioners to help tackle the causes of human error in the context of maritime accidents were suggested.

The implications of this study are several. First, the proposed agenda for future researchers can advance the field of human error in the maritime transport context by providing different theoretical perspectives and adapting research methods from social and human sciences. Second, this study highlights the gap in our current understanding of the role of human error in maritime accidents, which can feed into curricula for the education and training of maritime cadets, seafarers and other personnel. Finally, by understanding these gaps, maritime organisations and stakeholders can implement policies that will embed human factors more specifically with the ultimate objective of improving safety in maritime transportation.

## Data Availability

All data generated and analysed during this study are available through Web of Science and are included in this published article (please see references).

## Appendix

See Tables

**Table 6:** Cluster A: main topics discussed in articles

Total articles	Major Themes		
	Managerial/HR	Socio-technical use	Individual and cognitive
(73)	(27)	(9)	(37)
	Inter ship communication (speed, inadequate planning) Human and Organisational Factors (HOFs) Workplace routines, teamwork, group decision making Safety culture Dangerous working conditions Training and competence requirements Formal Safety Assessment (FSA) Bridge Resource Management (BRM) Leadership error No compliance with legislation on-board (Safety Management System – SMS)	Dynamic society Misuse of instruments (individual, organizational) Automation	Fatigue, stress, health Cognitive errors Situational Awareness (SA) Human Reliability Analysis (HRA) Human Factors Analysis and Classification System (HFACS) Cognitive Reliability and Error Analysis Method (CREAM)

6 and

**Table 7:** Cluster C: main topics discussed in articles

Total articles	Major Themes		
	Managerial/HR	Socio-technical use	Individual and cognitive
47	(10)	(14)	(23)
	Regulatory control Scheduling policy changes Quaternion Ship Domain (QSD)	Decoding, visualization and analysis of the AIS data Interpretation of Radar information for improved decision-making Marine Geographic Information System (MGIS) Autonomous Guidance and Navigation (AGN)	Human Factors Analysis and Classification System (HFACS) Marine Accident Risk Calculation System (MARCS) Cognitive Maps (CM) Quantitative risk assessment (QRA) Swiss cheese model Prince William Sound risk assessment Formal Safety Assessment (FSA)

7.

## Footnotes

1 In line with academic literature (Rothblum 2002:1) this paper refers to accidents and distinguishes between accidents and incidents depending on the severity of damage. Insurance company reports (SSR 2021) refer to any damage no matter the severity (including sinking of a vessel) as an “incident”.

2 (N.A. Stanton; G.H. Walker; P.H. Seong; S.A. Shappell; D.A. Wiegmann; S.W.A. Dekker; W. Jung; M.G. Lenne; S. Nazir, P. Waterson and J. Kim).

3 (E. Akyuz; J. Wang; M. Celik; F. Khan; Mosleh; R. Abassi).
